# The ErbB signalling pathway: protein expression and prognostic value in epithelial ovarian cancer

**DOI:** 10.1038/sj.bjc.6604471

**Published:** 2008-07-15

**Authors:** P de Graeff, A P G Crijns, K A ten Hoor, H G Klip, H Hollema, K Oien, J M Bartlett, G B A Wisman, G H de Bock, E G E de Vries, S de Jong, A G J van der Zee

**Affiliations:** 1 Department of Gynaecologic Oncology, University Medical Centre Groningen, University of Groningen, Hanzeplein 1, Groningen 9713 GZ, The Netherlands; 2 Department of Pathology, University Medical Centre Groningen, University of Groningen, Hanzeplein 1, Groningen 9713 GZ, The Netherlands; 3 Centre for Oncology and Applied Pharmacology, Cancer Research UK Beatson Laboratories, Garscube Estate, Switchback Road, Bearsden, Glasgow G61 1BD, UK; 4 Endocrine Cancer Group, Western General Hospital, Edinburgh Cancer Research Centre, University of Edinburgh, Crewe Road South, Edinburgh EH4 2XR, UK; 5 Department of Epidemiology and Statistics, University Medical Centre Groningen, University of Groningen, Hanzeplein 1, Groningen 9713 GZ, The Netherlands; 6 Department of Medical Oncology, University Medical Centre Groningen, University of Groningen, Hanzeplein 1, Groningen 9713 GZ, The Netherlands

**Keywords:** ovarian cancer, prognosis, EGFR, HER-2/neu, PTEN, EGFRvIII

## Abstract

Ovarian cancer is the most frequent cause of death from gynaecological cancer in the Western world. Current prognostic factors do not allow reliable prediction of response to chemotherapy and survival for individual ovarian cancer patients. Epidermal growth factor receptor (EGFR) and HER-2/neu are frequently expressed in ovarian cancer but their prognostic value remains unclear. In this study, we investigated the expression and prognostic value of EGFR, EGFR variant III (EGFRvIII), HER-2/neu and important downstream signalling components in a large series of epithelial ovarian cancer patients. Immunohistochemical staining of EGFR, pEGFR, EGFRvIII, Her-2/neu, PTEN (phosphatase and tensin homologue deleted on chromosome 10), total and phosphorylated AKT (pAKT) and phosphorylated ERK (pERK) was performed in 232 primary tumours using the tissue microarray platform and related to clinicopathological characteristics and survival. In addition, EGFRvIII expression was determined in 45 tumours by RT–PCR. Our results show that negative PTEN immunostaining was associated with stage I/II disease (*P*=0.006), non-serous tumour type (*P*=0.042) and in multivariate analysis with a longer progression-free survival (*P*=0.015). Negative PTEN staining also predicted improved progression-free survival in patients with grade III or undifferentiated serous carcinomas (*P*=0.011). Positive pAKT staining was associated with advanced-stage disease (*P*=0.006). Other proteins were expressed only at low levels, and were not associated with any clinicopathological parameter or survival. None of the tumours were positive for EGFRvIII. In conclusion, our results indicate that tumours showing negative PTEN staining could represent a subgroup of ovarian carcinomas with a relatively favourable prognosis.

Five-year survival of advanced-stage ovarian cancer patients remains only 15–25%, despite intensive surgical treatment and combination chemotherapy. Development of intrinsic or acquired resistance to platinum-containing chemotherapy is the major obstacle in the treatment of patients with ovarian cancer (Bhoola and Hoskins, 2006). Current clinicopathological prognostic factors do not allow individualised prediction of response to chemotherapy or disease outcome. Identification of molecular biological prognostic factors would be of great value for more accurately classification of ovarian carcinomas into subtypes with a different clinical outcome, thereby possibly also enabling individualised treatment strategies (Crijns *et al*, 2006a).

Epidermal growth factor receptor (EGFR) and HER-2/neu are members of the erbB family of tyrosine kinase receptors. Aberrant activity of EGFR and HER-2/neu has been shown to be important in tumour growth and development. Binding of ligand to the ectodomain of ErbB receptors results in receptor autophosphorylation and initiation of downstream signalling cascades, such as the PI3K/AKT pathway and the Ras/Raf/MEK/Erk pathway. Activation of these pathways in cancer has been associated with increased angiogenesis, metastasis, dedifferentiation, growth and protection from apoptosis (Yarden and Sliwkowski, 2001). Phosphatase and tensin homologue deleted on chromosome 10 (PTEN) directly antagonises the PI3K/AKT pathway by preventing the phosphorylation of AKT (Sansal and Sellers, 2004).

Several studies have shown that overexpression of HER-2/neu and EGFR, as well as alterations in their downstream targets AKT and extracellular signal-regulated kinase (ERK) is associated with resistance to platinum- and taxane-based chemotherapy. Treatment with agents directed against these proteins may enhance chemotherapy-induced cell death (Ciardiello *et al*, 2000; Altomare *et al*, 2004; Qiu *et al*, 2005; Lee *et al*, 2005b) The prognostic significance of EGFR and HER-2/neu has been extensively studied in ovarian cancer, but remains unclear. A recent meta-analysis revealed that abnormal expression of these markers appears to be associated with poor 5-year survival, but this is not a uniform finding (Crijns *et al*, 2003).

The EGFR variant III (EGFRvIII) lacks exons 2–7 of the extracellular domain of the receptor. Although EGFRvIII is unable to bind ligand, it is constitutively phosphorylated and able to activate downstream signalling pathways (Pedersen *et al*, 2001). EGFRvIII expression is thought to confer resistance to cisplatin and paclitaxel (Nagane *et al*, 1998; Montgomery *et al*, 2000). The two studies investigating EGFRvIII expression in ovarian cancer show conflicting results (Moscatello *et al*, 1995; Lassus *et al*, 2006).

The aim of the present study was to investigate the prognostic significance of EGFR and HER-2/neu, and their downstream targets AKT, ERK and PTEN in a large series of 232 epithelial ovarian cancer patients using the tissue microarray (TMA) platform. In addition to immunostaining, we determined the expression of EGFRvIII in more detail in a subset of 45 ovarian tumours using the more sensitive method RT–PCR.

## Materials and methods

### Patients

Since 1985 all clinicopathological and follow-up data of 329 epithelial ovarian cancer patients treated at the University Medical Centre Groningen have been prospectively stored in a database. All patients gave informed consent for data storage and tumour collection, and studies were conducted in accordance with the Declaration of Helsinki principles and institutional review board policies. For the current study all consecutive chemonaive ovarian cancer patients for whom sufficient paraffin-embedded tumour tissue and complete follow-up data were available were selected (*n*=232).

Patients were surgically staged according to FIGO (International Federation of Gynaecology and Obstetrics) criteria (Cancer Committee of the International Federation of Gynaecology and Obstetrics, 1986). Optimal and suboptimal debulking was defined as the largest residual tumour lesions having a diameter of <2 cm or⩾2 cm. The histology of all carcinomas was determined by a gynaecological pathologist according to WHO (World Health Organization) criteria (Scully, 2004).

Response to chemotherapy was evaluated according to WHO criteria (World Health Organization, 1979). When indicated, intervention surgery was performed after three cycles of chemotherapy, while until 1996 second-look surgery was regularly performed after six cycles of chemotherapy.

### TMA construction and immunostaining

Tissue microarrays were constructed as described previously (de Graeff *et al*, 2006). In total, four tissue cores from 232 primary tumours and 45 paired tumours obtained at second-look surgery or surgery for recurrent disease were included on eight TMAs.

Antigen retrieval methods, primary antibodies and detection techniques are provided as supplementary data. Sections (4 *μ*m) were de-paraffinised in xylene and endogenous peroxidase was blocked by incubation in 0.3% hydrogen peroxide for 30 min. After antigen retrieval, slides were incubated in normal goat serum (HER-2/neu), horse serum (EGFR, pEGFR), bovine serum (phosphorylated AKT (pAKT), phosphorylated ERK (pERK), PTEN, total AKT) or blocking solution (Dako, Cambridgeshire, UK) for EGFR. For pEGFR, pAKT, pERK and PTEN staining, endogenous avidin and biotin activity was blocked using a blocking kit (Vector Laboratories, Burlingame, UK). HER-2/neu staining was performed in a Dako autostainer (Dako). Staining was visualised by 3′3-diaminobenzidine tetrahydrochloride and sections were counterstained with haematoxylin. EGFRvIII staining was kindly performed by Dr A Jungbluth, Ludwig Institute for Cancer Research, New York, USA.

Positive controls included separate TMA slides containing multiple tumour and normal tissues for EGFR and pEGFR, sections from tumours with known marker expression for HER-2/neu and PTEN, ovarian cancer cell line A2780 for AKT, pAKT and ERK, and glioblastoma cell line U87 transfected with an EGFRvIII plasmid for EGFRvIII staining (Jungbluth *et al*, 2003). Negative controls were obtained by omission of the primary antibody, and by incubation with normal rabbit IgG for total AKT. All control experiments gave satisfactory results. Antigen preservation was verified by vimentin staining, which was positive in all tumour and control samples.

Evaluation of immunostaining was independently performed by two observers (KAH and PDG), blinded to clinical data. The agreement between the two observers was>90%. Discordant cases were reviewed with a gynaecological pathologist and were re-assigned on consensus of opinion.

HER-2/neu staining was scored according to the HercepTest protocol (Lebeau *et al*, 2001), and was considered positive when>10% of tumour cells showed moderate or strong membrane staining. For EGFR and EGFRvIII, tumours demonstrating>10% membrane staining were considered to show overexpression (Elie *et al*, 2004; Skirnisdottir *et al*, 2004; Cunningham *et al*, 2005). Overexpression of p-EGFR was defined as >5% membrane or granular cytoplasmic staining (Han *et al*, 2004). Tumours were considered positive for AKT or ERK if >10% of tumour cells showed positive cytoplasmic and/or nuclear staining (Kurose *et al*, 2001). Phosphatase and tensin homologue deleted on chromosome 10 staining in tumour sample was scored relative to staining in vascular endothelium (Gimm *et al*, 2000; Choe *et al*, 2003), and was regarded as negative when staining was completely absent in tumour tissue but present in vascular endothelium.

### RT–PCR for EGFRvIII

We performed RT–PCR analysis on a subset of 45 frozen tumour samples, of which 35 showed positive immunostaining for (p)EGFR or downstream targets and 10 were completely negative. Positive controls included a glioblastoma tumour sample expressing both the wild-type EGFR (wtEGFR) and EGFRvIII, and a cell line transfected with an EGFRvIII plasmid (Jurkat.EGFRvIII; Bremer *et al*, 2005).

Extraction of RNA and cDNA synthesis was performed as previously described (Crijns *et al*, 2006b). We performed RT–PCR separately for EGFRvIII and the housekeeping gene *GAPDH*. Primers were 5′-GGGCTCTGGAGGAAAAGAAA-3′ and 5′-AGGCCCTTCGCACTTCTTAC-3′ for amplifying EGFRvIII and wtEGFR (Ji *et al*, 2006), and 5′-CACCCACTCCTCCACCTTTG-3′ and 5′-CCACCACCCTGTTGCTGTAG-3′ for amplifying *GAPDH*. The protocol was as follows: initial denaturation at 95°C for 10 min, followed by 30 (EGFRvIII) or 25 cycles (*GAPDH*) of amplification (1 min at 95°C, 1 min at 56°C for EGFRvIII and at 60°C for *GAPDH*, and 90 s at 72°C) and a final extension step at 72°C for 7 min. The RT–PCR products (128 bp for EGFRvIII, 929 bp for wtEGFR and 110 bp for *GAPDH*) were visualised by 1.5% agarose gel electrophoresis in 1 × Tris-Borate EDTA buffer.

### Statistical analysis

Statistical analysis was carried out using the SPSS 12.01 software package. Cut-off points for positive marker expression were determined *a priori*. All cases with <2 evaluable cores were excluded from analysis.

Comparisons between paired tumour samples obtained before and after chemotherapy were made using the Wilcoxon rank sum test. Associations between markers, and between markers and clinicopathological characteristics were performed using the *χ*^2^ or Fisher's exact test, where appropriate.

The end points investigated were progression-free and disease-specific overall survival (PFS and OS), defined as the time from primary surgery until progression/relapse of the disease or death of ovarian cancer, respectively. Response to platinum-based chemotherapy could only be evaluated in patients who had measurable disease after primary surgery and/or during first-line chemotherapy (*n*=130), and was defined according to WHO criteria (World Health Organization, 1979).

For univariate and multivariate survival analysis Cox proportional hazards model was used. Categorised covariates that were significant in univariate analysis were entered simultaneously into the multivariate model. Response to chemotherapy was analysed using logistic regression analysis. For this analysis, response was entered as a categorical variable (complete and partial response *vs* stable and progressive disease). *P*-values <0.05 were considered statistically significant.

## Results

### Patients

A total of 232 patients (median age 57.8 years, range 22–90) treated at the Groningen University Medical Centre between 1985 and 2002 were selected for the present study (Table 1). Of them 64 (27.6%) patients presented with stage I/II disease and 166 (71.5%) patients with stage III/IV disease. Optimal debulking was achieved in 61 (96.8%) stage I/II patients and 48 (31.0%) stage III/IV patients. First-line chemotherapy regimens were platinum based in 100 (43.1%) patients and platinum- and taxane-based in 72 (31.0%) patients. Total 25 (10.8%) patients were treated with other regimens, and 32 (13.8%) patients did not receive chemotherapy because of stage Ia disease, comorbidity or treatment refusal.

For stage I/II patient, 5-year PFS was 73.0% (median 53 months, range 0–207) and 5-year OS was 78.9% (median 58 months, range 0–207). For stage III/IV patients, 5-year PFS was 13.8% (median 13.8 months, range 0–149) and 5-year OS was 22.3% (median 21 months, range 0–213). Five-year survival for the whole cohort was 39.2%.

### Immunostaining and RT–PCR

**Figure 1:**
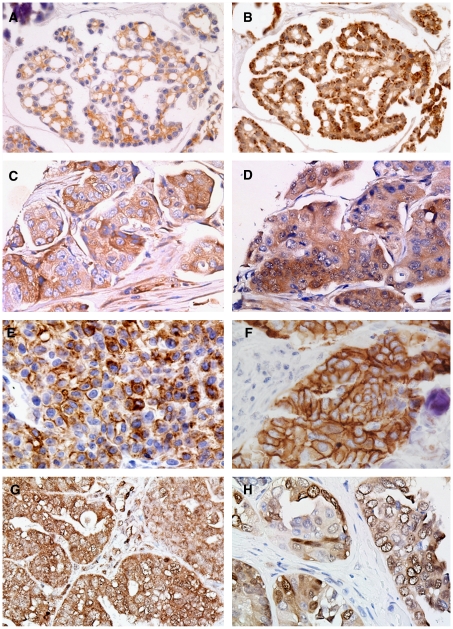
Results of immunostaining. (**A**) and (**B**) show positive immunostaining for epidermal growth factor receptor (EGFR) and pEGFR, respectively, in the same tumours. Positive immunostaining for pAKT and phosphatase and tensin homologue deleted on chromosome 10 (PTEN) in the same tumour is shown in (**C**) and (**D**), respectively. Figures (**E**–**G**) show positive immunostaining for EGFRvIII (positive control, **E**), HER-2/neu (**F**), pERK (**G**) and total AKT (**H**).

The number of non-evaluable primary tumours due to core loss during staining procedures or absence of tumour tissue ranged from 2 (0.9%) for HER-2/neu staining to 10 (4.3%) for pERK staining. Positive staining was present in 6.2% of tumours for EGFR, 5.1% of tumours for HER-2/neu, 11.8% tumours for pEGFR, 100% of tumours for total AKT, 8.3% of tumours for pAKT and 36.9% of tumours for pERK (Table 2; Figure 1). Of 224 tumours, 69 (30.8%) showed completely negative PTEN staining. None of the tumour samples stained positive for EGFRvIII, nor could EGFRvIII be detected by RT–PCR. Staining for pERK was more frequent in tumour samples obtained after three or six cycles of chemotherapy compared to paired primary tumour samples (65 *vs* 37%, *P*=0.020). For all other proteins, staining patterns in primary tumours were comparable to paired residual or recurrent tumour samples (Table 2).

Unexpectedly, PTEN staining was positively correlated with pAKT staining (*P*=0.034). No associations were found between other proteins (data not shown).

### Clinicopathological characteristics

Overexpression of EGFR was more frequent in non-serous tumours (*P*=0.017; Table 3). Stage III/IV tumours more often showed overexpression of pAKT (*P*=0.029). Loss of PTEN was related to stage I/II disease (*P*=0.006). Furthermore, negative PTEN immunostaining was associated with non-serous tumour type (*P*=0.042), occurring in 25% of serous, 39% of endometrioid, 42% of mucinous and 56% of clear cell tumours. No other associations between protein expression and clinicopathological variables were found.

### Response to chemotherapy and survival

Univariate Cox regression analysis revealed that patients with a PTEN-negative tumour had a better PFS and OS (Table 4; *P*<0.001 and *P*=0.037, respectively). On the basis of recent publications dividing ovarian carcinomas into subgroups with specific molecular alterations (Bell, 2005; Press *et al*, 2008), we performed subgroup analyses for early and late stage patients, and for patients with grade III and undifferentiated carcinomas. Subgroup analysis for stage I/II and stage III/IV patients showed that PTEN predicts PFS only in the early stage group (HR 0.29, 95% CI 0.095–0.9, *P*=0.032 for stage I/II patients, HR 0.74, 95% CI 0.48–1.15, *P*=0.18 for stage III/IV patients). Loss of PTEN also predicted improved PFS in 91 poorly differentiated serous carcinomas, of which 20 (22.0%) were PTEN negative (HR 0.43, 95% CI 0.23–0.83, *P*=0.011).

In multivariate analysis PTEN staining (*P*=0.015), FIGO stage (*P*=0.013) and residual tumour after primary surgery (*P*<0.001) independently predicted PFS (Table 5). Tumour stage (*P*=0.023) and residual tumour (*P*<0.001), but not PTEN staining (*P*=0.833) were significant prognostic factors in multivariate analysis for OS. Other markers were not associated with survival. Protein expression did not predict response to platinum-based chemotherapy.

## Discussion

Our study in a large, well-defined series of epithelial ovarian cancer patients shows that PTEN-negative tumours might represent a subgroup of ovarian carcinomas with a relatively favourable prognosis. To our knowledge this is the first study describing a relationship between negative PTEN staining and improved survival in ovarian cancer. Although a relationship between negative PTEN staining and improved survival has been described for endometrial cancer patients (Risinger *et al*, 1998), previous studies in ovarian cancer found no or an inverse relationship between PTEN and prognosis (Schondorf *et al*, 2003; Wang *et al*, 2005; Lee *et al*, 2005a). These contrasting results could be explained by the fact that previous studies either did not have the power to evaluate possible relations with survival, or restricted their analysis to stage III/IV ovarian cancer patients. In the current study PTEN staining was of prognostic significance mainly in the stage I/II group and in poorly differentiated serous carcinomas.

We found negative PTEN expression in 30.8% of tumours, which is in agreement with previous studies (Wang *et al*, 2005; Lee *et al*, 2005a; Hashiguchi *et al*, 2006). In ovarian cancer, loss-of-heterozygosity (LOH) at the PTEN locus (10q23.3) occurs in 31–45% of tumours, whereas mutations of the second PTEN allele are relatively rare (Maxwell *et al*, 1998; Obata *et al*, 1998; Kurose *et al*, 2001). Loss of protein expression is therefore also thought to arise through other mechanisms, such as DNA methylation (Sansal and Sellers, 2004).

Interestingly, we showed a high rate of negative PTEN staining in endometrioid and clear cell tumours. A high rate of PTEN loss in clear cell and endometrioid carcinomas has also been shown in previous, much smaller studies (Obata *et al*, 1998; Hashiguchi *et al*, 2006). Both cancers are thought to at least partly arise from endometriosis. Sato *et al* (2000) showed that in three out of five ovarian carcinomas associated with endometriosis, LOH at 10q23.3 occurs in both the carcinoma and in endometriotic lesions, implicating that LOH is an early event in carcinogenesis and that PTEN is involved in the progression from endometriotic precursor lesion to clear cell or endometrioid ovarian cancer.

Our results show that negative PTEN staining is strongly associated with early stage disease and a non-serous tumour type. Recent studies suggest that ovarian carcinomas could be divided in two categories. The first category, called type I, includes low-grade serous, mucinous, clear cell and endometrioid tumour with frequent alterations in BRAF, KRAS and PTEN. Type I tumours are thought to arise from precursor lesions such as endometriosis and have a relatively good prognosis. In contrast, type II tumours, including high-grade serous and undifferentiated carcinomas characterised by p53 mutations and overexpression/amplification of HER-2/neu and AKT2, tend to show a highly aggressive behaviour (Shih and Kurman, 2004; Bell, 2005). In the present study, we identified a relationship of pAKT expression with late stage disease. Moreover, our previous work showed that overexpression of p53 mostly occurs in high-grade, late stage, serous carcinomas (de Graeff *et al*, 2006). Our combined results therefore support this model of ovarian carcinogenesis.

A recent study by Press *et al* (2008) suggests that type II ovarian tumours can be subclassified into three groups based on their BRCA1 status. Their results indicate that poorly differentiated serous carcinomas with BRCA1 mutations frequently show loss of PTEN. The molecular mechanism underlying the relationship between loss of PTEN and BRCA1 mutations in ovarian cancer remains unknown. Possibly, ineffective DNA repair in BRCA1-linked tumours results in specific mutations of the *PTEN* gene (Foulkes, 2008; Saal *et al*, 2008). On the basis of these observations we performed survival analysis in a subgroup of 91 poorly differentiated serous carcinomas. We were able to show that loss of PTEN was indeed associated with improved PFS in this subgroup of ovarian carcinomas. Patients with BRCA1-linked hereditary tumours have a favourable survival compared to sporadic tumours, possibly because of a good response to chemotherapy (Boyd *et al*, 2000; Chetrit *et al*, 2008). The link between PTEN and BRCA1 status might therefore explain an improved disease outcome in a subgroup of patients with an otherwise very poor prognosis. In that case, IHC staining of PTEN may be a rapid way of identifying tumours most likely to carry BRCA1 mutations. Subsequently, those patients might benefit from treatments with agents selectively targeting BRCA mutant tumour cells, such as poly(ADP-ribose) polymerase 1 inhibitors (Farmer *et al*, 2005).

In the current study, loss of PTEN was associated with improved PFS, but not OS. As PFS is closely related to response to chemotherapy, these results might indicate that patients with PTEN negative tumours respond favourably to first-line therapy. In the current study we did not observe a relationship between PTEN status and response to chemotherapy. However, this analysis was limited to patients who had measurable disease before start of chemotherapy or measurable disease progression during treatment. Response to chemotherapy could therefore only be analysed in a subset of advanced-stage patients with a very poor prognosis. One possible explanation for the lack of association between negative PTEN staining and OS might be explained by the fact that tumours can acquire secondary mutations during or after platinum-based chemotherapy (Sakai *et al*, 2008). Once a patient presents with progressive or recurrent disease, these mutations may render the tumour insensitive to platinum-based chemotherapy irrespective of the PTEN status.

We did not observe any association between EGFR and HER-2/neu immunostaining and disease outcome, confirming results of a previous study also from our institution (Van Der Zee *et al*, 1995). Previous studies on the relationship between EGFR or HER-2/neu overexpression and clinicopathological characteristics, response to chemotherapy and survival have shown conflicting results (Camilleri-Broet *et al*, 2004; Elie *et al*, 2004; Nielsen *et al*, 2004; Psyrri *et al*, 2005). One of the most important reasons for these inconclusive data is the considerable methodological variability among studies (Hall *et al*, 2004). Techniques used to determine marker expression, antibodies and scoring systems used for immunostaining vary widely between studies. For the present investigation, we aimed to use well-characterised antibodies that have been extensively studied in other tumour types, and, if possible, used well-defined scoring criteria that have been shown to be reproducible. We have sought to adhere to the REMARK guidelines for publishing prognostic factor studies (McShane *et al*, 2005). The use of these guidelines and of standardised methods should aid in increasing transparency and reproducibility of prognostic factor studies in ovarian cancer and other tumour types.

As tumours showing evidence of strong signalling through a particular pathway are thought to have a high chance of responding to therapies directed against this pathway, the identification of reliable biomarkers could aid in selecting patients who are most likely to benefit from targeted therapy (Bild *et al*, 2006). Results of different clinical trials show that positive immunostaining for HER-2/neu or EGFR does not reliably predict response to ErbB-targeted therapy (Ciardiello and Tortora, 2008). A possible better marker of response to EGFR- and HER-2/neu-targeted therapies is activation or downregulation of downstream pathways. Indeed, positive immunostaining for pAKT, pERK, PTEN and EGFRvIII has been reported to predict sensitivity to EGFR tyrosine kinase inhibitors in non-small-cell lung cancer and glioblastoma (Han *et al*, 2004; Mellinghoff *et al*, 2005). The association of pAKT and pERK in relation to response to ErbB-targeted therapy in ovarian cancer has not been studied yet, but expression of these proteins might be used as a marker of responsiveness to targeted therapies. Our results show that 8.3 and 36.9% of tumours show positive pAKT and pERK staining, respectively, indicating that only a subgroup of patients might benefit from agents directed against these pathways. As pERK is overexpressed in approximately one-third of primary ovarian tumours and 65% of tumour samples from primary chemo-resistant tumours obtained after chemotherapy, treatment of patients with Ras/Raf/MEK/Erk-targeted agents appears to be an interesting therapeutic option (Messersmith *et al*, 2006).

In contrast to previous studies, we show a low percentage of pAKT-positive tumours (Altomare *et al*, 2004; Wang *et al*, 2005). The discrepancy between our results and those obtained in previous studies is not likely to be due to methodological variability. We have used the same well-characterised antibody that was used in previous studies, with a comparable staining protocol. In all our experiments, the ovarian cancer cell line A2780 served as a positive control. Expression of pAKT in this cell line was confirmed by western blotting (data not shown). In agreement with previous large studies, we also show a relatively low percentage of EGFR- and HER-2/neu-overexpressing tumours (Bookman *et al*, 2003; Lassus *et al*, 2006). We therefore conclude that in this group of ovarian carcinomas, signalling of EGFRs via the AKT pathway might be important only in specific subgroups of ovarian tumours.

Surprisingly, we identified a significant relationship between positive expression of AKT and positive expression of PTEN. The role of PTEN as a negative regulator of AKT is well documented in both cell line models and tumour samples (Stambolic *et al*, 1998; Sun *et al*, 1999; Kurose *et al*, 2001; Choe *et al*, 2003). However, others have also identified a positive correlation between expressions of the two proteins by immunostaining (Panigrahi *et al*, 2004; Slipicevic *et al*, 2005; Wang *et al*, 2005). This might mean that in tumours, the regulatory relationship between AKT and PTEN is not linear. In breast and ovarian cancer, it has been shown that aberrations of the *PI3K* and *PTEN* genes are mutually exclusive (Saal *et al*, 2005; Press *et al*, 2008), resulting in constitutive activation of the PI3K pathway in the presence of an intact PTEN. Loss of PTEN may also contribute to tumourigenesis and progression via AKT-independent pathways, such as the p53 pathway (Blanco-Aparicio *et al*, 2007).

In contrast to available data in literature we did not detect any EGFRvIII in this large group of ovarian carcinomas. Moscatello *et al* reported that EGFRvIII is expressed in 75% of ovarian tumours, but this high percentage could not be confirmed in subsequent studies (Jungbluth *et al*, 2003; Lassus *et al*, 2006). We determined EGFRvIII status by immunohistochemistry using the well-defined antibody DH8.3 and verified our results at the RNA level by RT–PCR on a subset of 45 tumours showing positive immunostaining for EGFR or downstream targets. As EGFRvIII heterodimerises with wtEGFR, is constitutively phosphorylated and activates AKT and to a lesser extent ERK, we hypothesised that the chance of finding EGFRvIII-positive tumours was largest in this subgroup (Montgomery *et al*, 1995; Li *et al*, 2004; Luwor *et al*, 2004). As we did not detect any EGFRvIII positivity in this subgroup, nor in 10 tumours that did not overexpress any of the studied markers, our data strongly suggest that EGFRvIII signalling does not play a major role in ovarian cancer.

In the current retrospective study we investigated protein expression in a large well-defined patient population. However, our results showed that protein expression was mainly important in specific patient groups. Unfortunately, these subgroups were too small to perform valid multivariate analysis. Furthermore, not all patients received the same chemotherapeutic treatment. Future studies should determine the prognostic value of PTEN staining, especially in early stage patients and poorly differentiated serous tumours, in large prospective studies including homogeneously treated patients.

In summary, we demonstrated that negative PTEN staining is associated with favourable patient and tumour characteristics, and independently predicts improved PFS. The importance of pAKT and pERK expression as downstream markers of responsiveness to receptor tyrosine kinase-targeted therapies deserves to be evaluated in clinical trials. A better understanding of these pathways and their role in ovarian cancer will enable us to use targeted drugs more efficiently, and to identify (groups of) genes that predict prognosis more accurately.

## Figures and Tables

**Table 1:** Clinicopathological characteristics

	**All patients (*n*=232)**	
	***N***	**%**
*FIGO stage*		
Stage I	45	19.4
Stage II	19	8.2
Stage III	133	57.3
Stage IV	33	14.2
Missing	2	0.9
		
*Tumour type*		
Serous	129	55.6
Mucinous	27	11.6
Clear cell	17	7.3
Endometrioid	33	14.2
Adenocarcinoma NOS	9	3.9
Other	17	7.3
		
*Tumour grade*		
Grade I	39	16.8
Grade II	51	22.0
Grade III	104	44.8
Undifferentiated	14	6.0
Missing	24	10.3
		
*Residual disease*		
<2 cm	111	47.8
⩾2 cm	109	47.0
Missing	12	5.2
		
*Type of chemotherapy*		
No chemotherapy	32	13.8
Platinum based	100	43.1
Platinum/taxane based	72	31.0
Other regimen	25	10.8
Missing	3	1.3

FIGO= International Federation of Gynaecology and Obstetrics; NOS=not otherwise specified.

**Table 2:** Results of immunostaining

	**EGFR**	**pEGFR**	**HER-2/neu**	**pAKT**	**pERK**	**PTEN**
*Primary tumours (*n=*232)*						
Evaluable^a^	228	228	230	228	222	224
Positive	16 (7.0%)	27 (11.8%)	12 (5.2%)	19 (8.3%)	82 (36.9%)	155 (69.2%)
Negative	212 (93.0%)	201 (88.2%)	218 (94.8%)	209 (91.7%)	140 (63.1%)	69 (30.8%)
						
*Second look (*n=*26)*						
Evaluable^a^	22	22	22	21	20	19
Positive	4 (18.2%)	5 (22.7%)	1 (4.5%)	4 (19.0%)	13 (65.0%)	16 (84.2%)
Negative	18 (81.8%)	17 (77.3%)	21 (95.5%)	17 (81.0%)	7 (35.0%)	3 (15.8%)
*P*-value^b^	0.317	0.317	1.000	0.317	**0.020**	0.655
						
*Recurrent disease (*n=*19)*						
Evaluable^a^	19	19	18	18	19	18
Positive	2 (10.5%)	3 (15.8%)	2 (11.1%)	3 (16.7%)	8 (42.1%)	17 (94.4%)
Negative	17 (89.5%)	16 (84.2%)	16 (88.9%)	15 (83.3%)	11 (57.9%)	1 (5.6%)
*P*-value^c^	0.317	0.564	0.157	0.317	0.317	0.317

Bold signifies *P*<0.05.

a Number of evaluable cases (cases with <2 evaluable cores were excluded from the analysis).

b *P*-value from Wilcoxon rank sum test for comparison of protein expression between tumour samples from primary surgery and from second look.

c *P*-value from Wilcoxon rank sum test for comparison of protein expression between tumour samples from primary surgery and surgery for recurrent disease.

**Table 3:** Relationship between proteins and clinicopathological characteristics

	**HER2**			**EGFR**			**pEGFR**			**pAKT**			**pERK**			**PTEN**		
**Variable**	**Neg**	**Pos**	***P***	**Neg**	**Pos**	***P***	**Neg**	**Pos**	***P***	**Neg**	**Pos**	***P***	**Neg**	**Pos**	***P***	**Neg**	**Pos**	***P***
*Age*																		
<58 years	110/113	3/113	0.14	106/112	6/112	0.44	99/113	14/113	0.84	104/114	10/114	1.00	69/108	39/108	0.89	38/111	73/111	0.31
>58 years	108/117	9/117		106/116	10/116		102/115	13/115		105/114	9/114		71/114	43/114		31/113	82/113	
																		
*Stage*																		
Early	59/62	3/62	1.00^*^	56/61	5/61	0.77^*^	51/60	9/60	0.49	60/91	1/61	0.029	38/59	21/59	0.88	27/60	33/60	0.006
Late	157/166	9/166		154/165	11/165		148/166	18/166		147/165	18/165		101/161	60/161		41/162	121/162	
																		
*Histology*																		
Serous	123/128	5/128	0.38	123/127	4/127	0.017	111/127	16/127	0.84	115//126	11/126	1.00	77/123	46/123	0.89	31/124	93/124	0.042
Other	95/102	7/102		89/101	12/101		90/101	11/101		94/102	8/102		63/99	36/99		38/100	62/100	
																		
*Grade*																		
I/II	84/89	5/89	1.00^*^	82/87	5/87	0.78	75/88	13/88	0.18	85/89	4/89	0.19	49/85	36/85	0.24	32/87	55/87	0.17
III/undiff	112/118	6/118		109/118	9/118		107/117	10/117		104/116	12/116		76/114	38/114		31/114	83/114	
																		
*Res. tumour*																		
<2 cm	105/109	4/109	0.54	100/107	7/107	1.00	95/107	12/107	0.84	102/108	6/108	0.31	70/102	32/102	0.15	34/104	70/104	0.55
⩾ 2 cm	102/109	7/109		101/109	8/109		95/109	14/109		97/108	11/108		63/108	45/108		31/108	77/108	

Neg=negative; Pos=positive; Res. tumour=residual tumour after primary surgery; undiff=undifferentiated.

*P*-values are derived from the *χ*^2^-test or Fischer's exact test, where appropriate (^*^signifies the use of the Fischer's exact test).

**Table 4:** Results of univariate survival analysis

	**Univariate Cox regression analysis**		
	**Hazard ratio**	**95% confidence interval**	***P*-value**
*Progression-free survival*			
EGFR positive	0.55	0.26–1.17	0.12
HER-2/neu positive	0.98	0.46–2.10	0.96
pEGFR positive	0.62	0.35–1.06	0.09
pAKT positive	0.88	0.46–1.67	0.69
pERK positive	1.09	0.77–1.54	0.64
PTEN negative	**0.48**	**0.32**–**0.72**	**<0.001**
			
*Overall survival*			
EGFR positive	0.84	0.43–1.65	0.43
HER-2/neu positive	1.02	0.48–2.20	0.94
pEGFR positive	0.64	0.36–1.39	0.13
pAKT positive	1.05	0.58–1.91	0.86
pERK positive	1.04	0.73–1.48	0.84
PTEN negative	**0.66**	**0.44**–**0.097**	**0.037**

Bold signifies *P*<0.05.

**Table 5:** Results of multivariate survival analysis

	**Multivariate Cox regression analysis**		
	**Hazard ratio**	**95% confidence interval**	***P***-**value**
*Progression-free survival*			
PTEN-negative tumour	**0.57**	**0.36**–**0.90**	**0.015**
Age>58 years	1.09	0.74–1.60	0.671
FIGO stage III/IV	**2.51**	**1.21**–**5.19**	**0.013**
Serous tumour type	1.44	0.92–2.24	0.109
Differentiation grade III/IV	1.40	0.89–2.19	0.144
Suboptimal debulking	**2.37**	**1.43**–**3.50**	**<0.001**
			
*Overall survival*			
PTEN-negative tumour	0.96	0.62–1.47	0.833
Age>58 years	1.24	0.83–1.83	0.291
FIGO stage III/IV	**2.56**	**1.14**–**5.74**	**0.023**
Serous tumour type	1.46	0.93–2.76	0.100
Differentiation grade III/IV	1.50	0.94–2.38	0.090
Suboptimal debulking	**2.51**	**1.57**–**4.00**	**<0.001**

PTEN=phosphatase and tensin homologue deleted on chromosome 10; FIGO=International Federation of Gynaecology and Obstetrics.

Bold signifies *P*<0.05.

## References

[bib1] Altomare DA, Wang HQ, Skele KL, De Rienzo A, Klein-Szanto AJ, Godwin AK, Testa JR (2004) AKT and mTOR phosphorylation is frequently detected in ovarian cancer and can be targeted to disrupt ovarian tumor cell growth. Oncogene 23: 5853–5857 15208673 10.1038/sj.onc.1207721

[bib2] Bell DA (2005) Origins and molecular pathology of ovarian cancer. Mod Pathol 18(Suppl 2): S19–S32 15761464 10.1038/modpathol.3800306

[bib3] Bhoola S, Hoskins WJ (2006) Diagnosis and management of epithelial ovarian cancer. Obstet Gynecol 107: 1399–1410 16738170 10.1097/01.AOG.0000220516.34053.48

[bib4] Bild AH, Potti A, Nevins JR (2006) Linking oncogenic pathways with therapeutic opportunities. Nat Rev Cancer 6: 735–741 16915294 10.1038/nrc1976

[bib5] Blanco-Aparicio C, Renner O, Leal JF, Carnero A (2007) PTEN, more than the AKT pathway. Carcinogenesis 28: 1379–1386 17341655 10.1093/carcin/bgm052

[bib6] Bookman MA, Darcy KM, Clarke-Pearson D, Boothby RA, Horowitz IR (2003) Evaluation of monoclonal humanized anti-HER2 antibody, trastuzumab, in patients with recurrent or refractory ovarian or primary peritoneal carcinoma with overexpression of HER2: a phase II trial of the Gynecologic Oncology Group. J Clin Oncol 21: 283–290 12525520 10.1200/JCO.2003.10.104

[bib7] Boyd J, Sonoda Y, Federici MG, Bogomolniy F, Rhei E, Maresco DL, Saigo PE, Almadrones LA, Barakat RR, Brown CL, Chi DS, Curtin JP, Poynor EA, Hoskins WJ (2000) Clinicopathologic features of BRCA-linked and sporadic ovarian cancer. JAMA 283: 2260–2265 10807385 10.1001/jama.283.17.2260

[bib8] Bremer E, Samplonius DF, van Genne L, Dijkstra MH, Kroesen BJ, de Leij LF, Helfrich W (2005) Simultaneous inhibition of epidermal growth factor receptor (EGFR) signaling and enhanced activation of tumor necrosis factor-related apoptosis-inducing ligand (TRAIL) receptor-mediated apoptosis induction by an scFv:sTRAIL fusion protein with specificity for human EGFR. J Biol Chem 280: 10025–10033 15644326 10.1074/jbc.M413673200

[bib9] Camilleri-Broet S, Hardy-Bessard AC, Le Tourneau A, Paraiso D, Levrel O, Leduc B, Bain S, Orfeuvre H, Audouin J, Pujade-Lauraine E (2004) HER-2 overexpression is an independent marker of poor prognosis of advanced primary ovarian carcinoma: a multicenter study of the GINECO group. Ann Oncol 15: 104–112 14679128 10.1093/annonc/mdh021

[bib10] Cancer Committee of the International Federation of Gynaecology and Obstetrics (1986) Staging announcement: FIGO Cancer Committee. Gynecol Oncol 25: 383–385

[bib11] Chetrit A, Hirsh-Yechezkel G, Ben-David Y, Lubin F, Friedman E, Sadetzki S (2008) Effect of BRCA1/2 mutations on long-term survival of patients with invasive ovarian cancer: the national Israeli study of ovarian cancer. J Clin Oncol 26: 20–25 18165636 10.1200/JCO.2007.11.6905

[bib12] Choe G, Horvath S, Cloughesy TF, Crosby K, Seligson D, Palotie A, Inge L, Smith BL, Sawyers CL, Mischel PS (2003) Analysis of the phosphatidylinositol 3′-kinase signaling pathway in glioblastoma patients *in vivo*. Cancer Res 63: 2742–2746 12782577

[bib13] Ciardiello F, Caputo R, Bianco R, Damiano V, Pomatico G, De Placido S, Bianco AR, Tortora G (2000) Antitumor effect and potentiation of cytotoxic drugs activity in human cancer cells by ZD-1839 (Iressa), an epidermal growth factor receptor-selective tyrosine kinase inhibitor. Clin Cancer Res 6: 2053–2063 10815932

[bib14] Ciardiello F, Tortora G (2008) EGFR antagonists in cancer treatment. N Engl J Med 358: 1160–1174 18337605 10.1056/NEJMra0707704

[bib15] Crijns AP, Duiker EW, De Jong S, Willemse PH, Van Der Zee AG, De Vries EG (2006a) Molecular prognostic markers in ovarian cancer: toward patient-tailored therapy. Int J Gynecol Cancer 16(Suppl 1): 152–165 10.1111/j.1525-1438.2006.00503.x 16515584

[bib16] Crijns AP, Gerbens F, Plantinga AE, Meersma GJ, De Jong S, Hofstra RM, De Vries EG, Van Der Zee AG, de Bock GH, te Meerman GJ (2006b) A biological question and a balanced (orthogonal) design: the ingredients to efficiently analyze two-color microarrays with confirmatory factor analysis. BMC Genomics 7: 232 16968534 10.1186/1471-2164-7-232 PMC1590029

[bib17] Crijns APG, Boezen HM, Schouten JP, Arts HJG, Hofstra RMW, Willemse PHB, Vries de EGE, Van Der Zee AG (2003) Prognostic factors in ovarian cancer: current evidence and future prospects. Eur J Cancer S 1: 127–145

[bib18] Cunningham MP, Essapen S, Thomas H, Green M, Lovell DP, Topham C, Marks C, Modjtahedi H (2005) Coexpression, prognostic significance and predictive value of EGFR, EGFRvIII and phosphorylated EGFR in colorectal cancer. Int J Oncol 27: 317–325 16010411

[bib19] de Graeff P, Hall J, Crijns AP, de Bock GH, Paul J, Oien KA, ten Hoor KA, de Jong S, Hollema H, Bartlett JM, Brown R, van der Zee AG (2006) Factors influencing p53 expression in ovarian cancer as a biomarker of clinical outcome in multicentre studies. Br J Cancer 95: 627–633 16880779 10.1038/sj.bjc.6603300 PMC2360689

[bib20] Elie C, Geay JF, Morcos M, Le Tourneau A, Girre V, Broet P, Marmey B, Chauvenet L, Audouin J, Pujade-Lauraine E, Camilleri-Broet S (2004) Lack of relationship between EGFR-1 immunohistochemical expression and prognosis in a multicentre clinical trial of 93 patients with advanced primary ovarian epithelial cancer (GINECO group). Br J Cancer 91: 470–475 15226774 10.1038/sj.bjc.6601961 PMC2409858

[bib21] Farmer H, McCabe N, Lord CJ, Tutt AN, Johnson DA, Richardson TB, Santarosa M, Dillon KJ, Hickson I, Knights C, Martin NM, Jackson SP, Smith GC, Ashworth A (2005) Targeting the DNA repair defect in BRCA mutant cells as a therapeutic strategy. Nature 434: 917–921 15829967 10.1038/nature03445

[bib22] Foulkes WD (2008) BRCA1—sowing the seeds crooked in the furrow. Nat Genet 40: 8–9 18163127 10.1038/ng0108-8

[bib23] Gimm O, Perren A, Weng LP, Marsh DJ, Yeh JJ, Ziebold U, Gil E, Hinze R, Delbridge L, Lees JA, Mutter GL, Robinson BG, Komminoth P, Dralle H, Eng C (2000) Differential nuclear and cytoplasmic expression of PTEN in normal thyroid tissue, and benign and malignant epithelial thyroid tumors. Am J Pathol 156: 1693–1700 10793080 10.1016/s0002-9440(10)65040-7 PMC1876937

[bib24] Hall J, Paul J, Brown R (2004) Critical evaluation of p53 as a prognostic marker in ovarian cancer. Expert Rev Mol Med 2004: 1–20 10.1017/S1462399404007781 15147608

[bib25] Han SW, Hwang PG, Chung DH, Kim DW, Im SA, Kim YT, Kim TY, Heo DS, Bang YJ, Kim NK (2004) Epidermal growth factor receptor (EGFR) downstream molecules as response predictive markers for gefitinib (Iressa (R), ZD1839) in chemotherapy-resistant non-small cell lung cancer. Int J Cancer 113: 109–115 10.1002/ijc.20550 15386420

[bib26] Hashiguchi Y, Tsuda H, Inoue T, Berkowitz RS, Mok SC (2006) PTEN expression in clear cell adenocarcinoma of the ovary. Gynecol Oncol 101: 71–75 16290000 10.1016/j.ygyno.2005.09.047

[bib27] Ji H, Zhao X, Yuza Y, Shimamura T, Li D, Protopopov A, Jung BL, McNamara K, Xia H, Glatt KA, Thomas RK, Sasaki H, Horner JW, Eck M, Mitchell A, Sun Y, Al Hashem R, Bronson RT, Rabindran SK, Discafani CM, Maher E, Shapiro GI, Meyerson M, Wong KK (2006) Epidermal growth factor receptor variant III mutations in lung tumorigenesis and sensitivity to tyrosine kinase inhibitors. Proc Natl Acad Sci USA 103: 7817–7822 16672372 10.1073/pnas.0510284103 PMC1456806

[bib28] Jungbluth AA, Stockert E, Huang HJ, Collins VP, Coplan K, Iversen K, Kolb D, Johns TJ, Scott AM, Gullick WJ, Ritter G, Cohen L, Scanlan MJ, Cavenee WK, Old LJ (2003) A monoclonal antibody recognizing human cancers with amplification/overexpression of the human epidermal growth factor receptor. Proc Natl Acad Sci USA 100: 639–644 12515857 10.1073/pnas.232686499 PMC141049

[bib29] Kurose K, Zhou XP, Araki T, Cannistra SA, Maher ER, Eng C (2001) Frequent loss of PTEN expression is linked to elevated phosphorylated Akt levels, but not associated with p27 and cyclin D1 expression, in primary epithelial ovarian carcinomas. Am J Pathol 158: 2097–2106 11395387 10.1016/S0002-9440(10)64681-0 PMC1891985

[bib30] Lassus H, Sihto H, Leminen A, Joensuu H, Isola J, Nupponen NN, Butzow R (2006) Gene amplification, mutation, and protein expression of EGFR and mutations of ERBB2 in serous ovarian carcinoma. J Mol Med 84: 671–681 16607561 10.1007/s00109-006-0054-4

[bib31] Lebeau A, Deimling D, Kaltz C, Sendelhofert A, Iff A, Luthardt B, Untch M, Lohrs U (2001) Her-2/neu analysis in archival tissue samples of human breast cancer: comparison of immunohistochemistry and fluorescence *in situ* hybridization. J Clin Oncol 19: 354–363 11208826 10.1200/JCO.2001.19.2.354

[bib32] Lee JS, Choi YD, Choi C, Lee MC, Park CS, Min KW (2005a) Expression of PTEN in ovarian epithelial tumors and its relation to tumor behavior and growth. Anal Quant Cytol Histol 27: 202–210 16220831

[bib33] Lee S, Choi EJ, Jin C, Kim DH (2005b) Activation of PI3K/Akt pathway by PTEN reduction and PIK3CA mRNA amplification contributes to cisplatin resistance in an ovarian cancer cell line. Gynecol Oncol 97: 26–34 15790433 10.1016/j.ygyno.2004.11.051

[bib34] Li B, Yuan M, Kim IA, Chang CM, Bernhard EJ, Shu HK (2004) Mutant epidermal growth factor receptor displays increased signaling through the phosphatidylinositol-3 kinase/AKT pathway and promotes radioresistance in cells of astrocytic origin. Oncogene 23: 4594–4602 15077177 10.1038/sj.onc.1207602

[bib35] Luwor RB, Zhu HJ, Walker F, Vitali AA, Perera RM, Burgess AW, Scott AM, Johns TG (2004) The tumor-specific de2–7 epidermal growth factor receptor (EGFR) promotes cells survival and heterodimerizes with the wild-type EGFR. Oncogene 23: 6095–6104 15221011 10.1038/sj.onc.1207870

[bib36] Maxwell GL, Risinger JI, Tong B, Shaw H, Barrett JC, Berchuck A, Futreal PA (1998) Mutation of the PTEN tumor suppressor gene is not a feature of ovarian cancers. Gynecol Oncol 70: 13–16 9698466 10.1006/gyno.1998.5039

[bib37] McShane LM, Altman DG, Sauerbrei W, Taube SE, Gion M, Clark GM (2005) REporting recommendations for tumour MARKer prognostic studies (REMARK). Br J Cancer 93: 387–391 16106245 10.1038/sj.bjc.6602678 PMC2361579

[bib38] Mellinghoff IK, Wang MY, Vivanco I, Haas-Kogan DA, Zhu S, Dia EQ, Lu KV, Yoshimoto K, Huang JH, Chute DJ, Riggs BL, Horvath S, Liau LM, Cavenee WK, Rao PN, Beroukhim R, Peck TC, Lee JC, Sellers WR, Stokoe D, Prados M, Cloughesy TF, Sawyers CL, Mischel PS (2005) Molecular determinants of the response of glioblastomas to EGFR kinase inhibitors. N Engl J Med 353: 2012–2024 16282176 10.1056/NEJMoa051918

[bib39] Messersmith WA, Hidalgo M, Carducci M, Eckhardt SG (2006) Novel targets in solid tumors: MEK inhibitors. Clin Adv Hematol Oncol 4: 831–836 17143253

[bib40] Montgomery RB, Guzman J, O'Rourke DM, Stahl WL (2000) Expression of oncogenic epidermal growth factor receptor family kinases induces paclitaxel resistance and alters beta-tubulin isotype expression. J Biol Chem 275: 17358–17363 10749863 10.1074/jbc.M000966200

[bib41] Montgomery RB, Moscatello DK, Wong AJ, Cooper JA, Stahl WL (1995) Differential modulation of mitogen-activated protein (MAP) kinase/extracellular signal-related kinase kinase and MAP kinase activities by a mutant epidermal growth factor receptor. J Biol Chem 270: 30562–30566 8530489 10.1074/jbc.270.51.30562

[bib42] Moscatello DK, Holgado-Madruga M, Godwin AK, Ramirez G, Gunn G, Zoltick PW, Biegel JA, Hayes RL, Wong AJ (1995) Frequent expression of a mutant epidermal growth factor receptor in multiple human tumors. Cancer Res 55: 5536–5539 7585629

[bib43] Nagane M, Levitzki A, Gazit A, Cavenee WK, Huang HJ (1998) Drug resistance of human glioblastoma cells conferred by a tumor-specific mutant epidermal growth factor receptor through modulation of Bcl-XL and caspase-3-like proteases. Proc Natl Acad Sci USA 95: 5724–5729 9576951 10.1073/pnas.95.10.5724 PMC20446

[bib44] Nielsen JS, Jakobsen E, Holund B, Bertelsen K, Jakobsen A (2004) Prognostic significance of p53, Her-2, and EGFR overexpression in borderline and epithelial ovarian cancer. Int J Gynecol Cancer 14: 1086–1096 15571614 10.1111/j.1048-891X.2004.14606.x

[bib45] Obata K, Morland SJ, Watson RH, Hitchcock A, Chenevix-Trench G, Thomas EJ, Campbell IG (1998) Frequent PTEN/MMAC mutations in endometrioid but not serous or mucinous epithelial ovarian tumors. Cancer Res 58: 2095–2097 9605750

[bib46] Panigrahi AR, Pinder SE, Chan SY, Paish EC, Robertson JF, Ellis IO (2004) The role of PTEN and its signalling pathways, including AKT, in breast cancer; an assessment of relationships with other prognostic factors and with outcome. J Pathol 204: 93–100 15307142 10.1002/path.1611

[bib47] Pedersen MW, Meltorn M, Damstrup L, Poulsen HS (2001) The type III epidermal growth factor receptor mutation. Biological significance and potential target for anti-cancer therapy. Ann Oncol 12: 745–760 11484948 10.1023/a:1011177318162

[bib48] Press JZ, De LA, Boyd N, Young S, Troussard A, Ridge Y, Kaurah P, Kalloger SE, Blood KA, Smith M, Spellman PT, Wang Y, Miller DM, Horsman D, Faham M, Gilks CB, Gray J, Huntsman DG (2008) Ovarian carcinomas with genetic and epigenetic BRCA1 loss have distinct molecular abnormalities. BMC Cancer 8: 17 18208621 10.1186/1471-2407-8-17 PMC2245962

[bib49] Psyrri A, Kassar M, Yu Z, Bamias A, Weinberger PM, Markakis S, Kowalski D, Camp RL, Rimm DL, Dimopoulos MA (2005) Effect of epidermal growth factor receptor expression level on survival in patients with epithelial ovarian cancer. Clin Cancer Res 11: 8637–8643 16361548 10.1158/1078-0432.CCR-05-1436

[bib50] Qiu L, Di W, Jiang Q, Scheffler E, Derby S, Yang J, Kouttab N, Wanebo H, Yan B, Wan Y (2005) Targeted inhibition of transient activation of the EGFR-mediated cell survival pathway enhances paclitaxel-induced ovarian cancer cell death. Int J Oncol 27: 1441–1448 16211241

[bib51] Risinger JI, Hayes K, Maxwell GL, Carney ME, Dodge RK, Barrett JC, Berchuck A (1998) PTEN mutation in endometrial cancers is associated with favorable clinical and pathologic characteristics. Clin Cancer Res 4: 3005–3010 9865913

[bib52] Saal LH, Gruvberger-Saal SK, Persson C, Lovgren K, Jumppanen M, Staaf J, Jonsson G, Pires MM, Maurer M, Holm K, Koujak S, Subramaniyam S, Vallon-Christersson J, Olsson H, Su T, Memeo L, Ludwig T, Ethier SP, Krogh M, Szabolcs M, Murty VV, Isola J, Hibshoosh H, Parsons R, Borg A (2008) Recurrent gross mutations of the PTEN tumor suppressor gene in breast cancers with deficient DSB repair. Nat Genet 40: 102–107 18066063 10.1038/ng.2007.39 PMC3018354

[bib53] Saal LH, Holm K, Maurer M, Memeo L, Su T, Wang X, Yu JS, Malmstrom PO, Mansukhani M, Enoksson J, Hibshoosh H, Borg A, Parsons R (2005) PIK3CA mutations correlate with hormone receptors, node metastasis, and ERBB2, and are mutually exclusive with PTEN loss in human breast carcinoma. Cancer Res 65: 2554–2559 15805248 10.1158/0008-5472-CAN-04-3913

[bib54] Sakai W, Swisher EM, Karlan BY, Agarwal MK, Higgins J, Friedman C, Villegas E, Jacquemont C, Farrugia DJ, Couch FJ, Urban N, Taniguchi T (2008) Secondary mutations as a mechanism of cisplatin resistance in BRCA2-mutated cancers. Nature 451: 1116–1120 18264087 10.1038/nature06633 PMC2577037

[bib55] Sansal I, Sellers WR (2004) The biology and clinical relevance of the PTEN tumor suppressor pathway. J Clin Oncol 22: 2954–2963 15254063 10.1200/JCO.2004.02.141

[bib56] Sato N, Tsunoda H, Nishida M, Morishita Y, Takimoto Y, Kubo T, Noguchi M (2000) Loss of heterozygosity on 10q23.3 and mutation of the tumor suppressor gene PTEN in benign endometrial cyst of the ovary: possible sequence progression from benign endometrial cyst to endometrioid carcinoma and clear cell carcinoma of the ovary. Cancer Res 60: 7052–7056 11156411

[bib57] Schondorf T, Gohring UJ, Roth G, Middel I, Becker M, Moser N, Valter MM, Hoopmann M (2003) Time to progression is dependent on the expression of the tumour suppressor PTEN in ovarian cancer patients. Eur J Clin Invest 33: 256–260 12641545 10.1046/j.1365-2362.2003.01116.x

[bib58] Scully RE (ed) (2004) Histological typing of ovarian tumours. In International Histological Classification of Tumours, World Health Organization, pp 11–19. Springer: Berlin

[bib59] Shih I, Kurman RJ (2004) Ovarian tumorigenesis: a proposed model based on morphological and molecular genetic analysis. Am J Pathol 164: 1511–1518 15111296 10.1016/s0002-9440(10)63708-x PMC1615664

[bib60] Skirnisdottir I, Seidal T, Sorbe B (2004) A new prognostic model comprising p53, EGFR, and tumor grade in early stage epithelial ovarian carcinoma and avoiding the problem of inaccurate surgical staging. Int J Gynecol Cancer 14: 259–270 15086725 10.1111/j.1048-891X.2004.014209.x

[bib61] Slipicevic A, Holm R, Nguyen MT, Bohler PJ, Davidson B, Florenes VA (2005) Expression of activated Akt and PTEN in malignant melanomas: relationship with clinical outcome. Am J Clin Pathol 124: 528–536 16146807 10.1309/YT58WWMTA6YR1PRV

[bib62] Stambolic V, Suzuki A, de la Pompa JL, Brothers GM, Mirtsos C, Sasaki T, Ruland J, Penninger JM, Siderovski DP, Mak TW (1998) Negative regulation of PKB/Akt-dependent cell survival by the tumor suppressor PTEN. Cell 95: 29–39 9778245 10.1016/s0092-8674(00)81780-8

[bib63] Sun H, Lesche R, Li DM, Liliental J, Zhang H, Gao J, Gavrilova N, Mueller B, Liu X, Wu H (1999) PTEN modulates cell cycle progression and cell survival by regulating phosphatidylinositol 3,4,5,-trisphosphate and Akt/protein kinase B signaling pathway. Proc Natl Acad Sci USA 96: 6199–6204 10339565 10.1073/pnas.96.11.6199 PMC26859

[bib64] Van Der Zee AG, Hollema H, Suurmeijer AJ, Krans M, Sluiter WJ, Willemse PH, Aalders JG, De Vries EG (1995) Value of P-glycoprotein, glutathione *S*-transferase pi, c-erbB-2, and p53 as prognostic factors in ovarian carcinomas. J Clin Oncol 13: 70–78 7799045 10.1200/JCO.1995.13.1.70

[bib65] Wang Y, Kristensen GB, Helland A, Nesland JM, Borresen-Dale AL, Holm R (2005) Protein expression and prognostic value of genes in the erb-b signaling pathway in advanced ovarian carcinomas. Am J Clin Pathol 124: 392–401 16191507 10.1309/BL7E-MW66-LQX6-GFRP

[bib66] World Health Organization (1979) Handbook for Reporting Results of Cancer Treatment. WHO: Geneva

[bib67] Yarden Y, Sliwkowski MX (2001) Untangling the ErbB signalling network. Nat Rev Mol Cell Biol 2: 127–137 11252954 10.1038/35052073

