# Supplementary material for: The ErbB signalling pathway: protein expression and prognostic value in epithelial ovarian cancer
Source: Br J Cancer. 2008 Jul 15;99(2):341–9. doi: 10.1038/sj.bjc.6604471 (PMC2480978; doi:10.1038/sj.bjc.6604471)
Supplement: Supplementary data [file 6604471x1.doc]

## Supplementary data: Antibodies used for immunohistochemical staining

| **Antigen** | **Antigen retrieval** | **Primary Antibody** | **Company** | **Dilution** | **Incubation time** | **Detection method** |
| --- | --- | --- | --- | --- | --- | --- |
| HER-2/neu | Tris/EDTA (pH8)^1^ | NCL-CBE-356 | Novocastra^3^ | 1:200 | 30 minutes | DAKO EnVision+ |
| EGFR | Trypsin digestion | 31G7 | Zymed^4^ | 1:50 | 60 minutes | DAKO Universal LSAB^TM^ Kit |
| pEGFR | Citrate (pH 6)^1^ | 1H12 | Cell Signaling^5^ | 1:500 | Overnight | DAKO Universal LSAB^TM^ Kit |
| AKT 1/2 | Autoclave^2^ | N-19 | Santa Cruz^6^ | 1:100 | Overnight | DAKO EnVision+ |
| pAKT 1/2 | Citrate (pH 6)^1^ | 736E11 | Cell signaling^5^ | 1:50 | Overnight | Avidin / Biotin |
| pERK 1/2 | Citrate (pH 6)^1^ | 20G11 | Cell Signaling^5^ | 1:50 | Overnight | Avidin / Biotin |
| PTEN | Citrate (pH 6)^1^ | 6H2.1 | Cascade^7^ | 1:50 | 60 minutes | Avidin / Biotin |
| EGFRvIII | EDTA (pH 8)^1^ | DH8.3 | 8 | 1 μg/μl | Overnight | Powervision HRP Plus System |

1. Sections were boiled in a microwave for 15 (HER-2/neu, pEGFR, pAkt, pErk, PTEN) or 45 (EGFRvIII) minutes;
2. 3 times 5 minutes at 155°C in blocking reagent (2% block + 0.2% SDS in maleic acid, pH 6.0, Boehringer Mannheim, Mannheim, Germany)
3. Novocastra, Newcastle upon Tyne, UK
4. Zymed, San Francisco, USA
5. Cell Signaling, Danvers, USA
6. Santa Cruz Biotechnology, Santa Cruz, USA
7. Cascade Bioscience, Winchester, USA
8. The DH8.3 antibody was kindly provided by dr. A Jungbluth (Ludwig Institute for Cancer Research, New York, USA).
